# Comparing Attitudes to Containment Measures of Patients, Health Care Professionals and Next of Kin

**DOI:** 10.3389/fpsyt.2018.00529

**Published:** 2018-10-26

**Authors:** Thomas Reisch, Simone Beeri, Georges Klein, Philipp Meier, Philippe Pfeifer, Etienne Buehler, Florian Hotzy, Matthias Jaeger

**Affiliations:** ^1^Hospital of Psychiatry Muensingen, Bern, Switzerland; ^2^University Hospital of Psychiatry and Psychotherapy, Bern, Switzerland; ^3^Département de Psychiatrie et Psychothérapie du Centre Hospitalier du Valais Romand, Monthey, Switzerland; ^4^Department for Psychiatry, Psychotherapy and Psychosomatics, University Hospital of Psychiatry Zurich, Zurich, Switzerland

**Keywords:** containment measures, coercive measures, coercion, fixation, intramuscular injection, mechanical restraint, physical

## Abstract

**Background:** In clinical psychiatric practice, health care professionals (HCP) must decide in exceptional circumstances after the weighing of interests, which, if any, containment measures including coercion are to be used. Here, the risk for patients, staff, and third parties, in addition to therapeutic considerations, factor into the decision. Patients' preference and the inclusion of relatives in these decisions are important; therefore, an understanding of how patients and next of kin (NOK) experience different coercive measures is crucial for clinical decision making. The aim of this study is to compare how patients, HCP, and NOK assess commonly used coercive measures.

**Methods:** A sample of 435 patients, 372 HCP, and 230 NOK completed the Attitudes to Containment Measures Questionnaire (ACMQ). This standardized self-rating questionnaire assessed the degree of acceptance or rejection of 11 coercive measures.

**Results:** In general, HCPs rated the coercive measures as more acceptable than did NOK and patients. The largest discrepancy in the ratings was found in regard to the application of coercive intramuscular injection of medication (effect size: 1.0 HCP vs. patients). However, the ratings by NOK were significantly closer to the patients' ratings compared to patients and HCP. The only exception was the acceptance of treatment in a closed acute psychiatric ward, which was deemed significantly more acceptable by NOK than by patients. Also, patients who had experienced coercive measures themselves more strongly refused other measures.

**Conclusion:** Patients most firmly rejected intramuscular injections, and the authors agree that these should only be used with reservation considering a high threshold. This knowledge about the discrepancy of the ratings should therefore be incorporated into professional training of HCP.

## Introduction

The implementation of coercive measures presents a major challenge for health care professionals (HCP). HCP face the dilemma of being responsible for safety while at the same time being obligated to promote therapy and take into account the self-determination and free will of the patient (1). Conversely, patients experience coercive measures as a “distinct negative incident” (2) and frequently as a traumatizing one (3). Coercive measures are usually applied to avert destructive actions against oneself, other patients, or staff. Aggression, especially assault of third persons, disorganization, and agitation, are common catalysts for coercive measures (4–7). Moreover, HCP often see the therapeutic effects of coercive measures (2, 8). Overall, the literature shows that some HCP see coercive measures as a necessary “emergency break” (9). However, several studies have demonstrated the negative effects of coercive measures, which have an unfavorable impact on the therapeutic relationship (10–13). In general, coercive measures lead to lower treatment satisfaction (14), reduce the effectiveness of the therapy (15), and prolong the duration of inpatient treatment (16). Still, it must be assumed that this is at least partly due to the fact that patients, especially those who experience coercive measures, often suffer from serious mental illness (17).

Coercive measures are exercised on a significant number of patients with strong differences between and within countries (18–20). In many countries, coercive measures are applied to 10–20% of all psychiatric inpatients (21–23). Significantly higher rates are reported in samples from other countries (19). In China, for example, 51.3% of all inpatients experienced coercive measures during their treatment; however, it must be noted that these international differences are related to variance in national legislation (20). Nonetheless, culture-specific attitudes and therapeutic approaches may also play a role in these major differences. In addition to differences in the absolute frequency of implementation between countries, the type of coercive measure applied also varies (23, 24). For example, in Germany, patients are more likely to be subject to mechanical restraint (23), a measure that is rarely used in English-speaking countries (25). In these countries, it is more common to physically restrain patients (26). Some countries, such as Switzerland (27) or the Netherlands (23), have high rates of seclusion.

Generally, a differentiation can be made between more and less invasive coercive measures. Measures, such as PRN (*pro re nata*) medication, observation, and time-out, are considered less invasive and are therefore preferable to the more invasive measures (28–30). However, particularly with violent patients, measures that more strongly limit personal freedom, including physical restraints, seclusion, and forced medication, may become inevitable (31).

Yet, the decision as to which of these more invasive measures is used is not rationally derivable but subject to the traditions of psychiatric clinics as well as legislation (22, 32). Researchers have pointed out that the patient's preference, often dependent on their previous experience with coercive measures, should be considered (33). Perceived coercion is an important mediating factor in the acceptance of coercive measures and is therefore, indirectly, responsible for the treatment outcome (34). When a higher level of coercion is perceived, patients feel powerless and inferior, and HCP suffer more guilt (35). These diverse perspectives provide context as to why the individual measures are perceived differently in various groups. Different perceptions of patients and HCP have been described in studies, especially in regard to forced intramuscular medication. While HCP were found to be more in favor of this measure, patients strongly rejected it (33, 36). Thus, it could be shown that patients who have experienced forced medication (orally or intramuscularly administered) also evaluated the treatment negatively 3 months later (disapproval of treatment) (37). A greater frequency of using forced medication also correlated with an increased negative evaluation of coercive measures (38). Differing ratings are also a factor in other forms of coercive measures. For example, patients have a significantly more negative assessment of the closed door of psychiatric wards than do HCP (39).

The involvement of NOK concerning the decision for coercive measures is considered standard today (40) (SAMW guideline). Research on ratings of coercive measures, however, is nearly non-existent. Ranieri et al. (41) showed that involuntary admission is perceived as less restrictive by NOK than by patients. This suggests that differences between NOK and patients in the ratings of specific coercive measures are to be expected.

In summary, there are varying perspectives, roles, and emotions of patients, HCP, and NOK regarding containment, especially coercive measures (2, 42–44). The acknowledgment of these differing attitudes is important for the therapeutic relationship and thus the treatment. The aim of this study is to highlight this very area of conflict, and the knowledge gained will be used to develop a better understanding to improve dialogue with patients (45, 46) and the training of HCP (44). Over time, such improvements could help reduce the stigma of psychiatry (10) and psychiatric clinics as safeguarding institutions (47).

Based on the cited literature and the previously mentioned considerations, we expect that HCP will generally show a higher acceptance of all coercive measures. Patients, however, will be more likely to reject coercive measures. We also expect that NOK will reject coercive measures less often than patients but more often than the HCP. In addition, we expect that the 3 samples will differ widely in their attitudes toward forced medication in particular. Patients who have experienced such a measure should, according to Dack, Ross, and Bowers (38), also evaluate other highly coercive measures more negatively.

## Method

### Data collection, in- and exclusion criteria, ethics, and anonymization

The study was conducted with 3 samples (patients, HCP, NOK) at 3 Swiss sites, i.e., the University Hospital of Psychiatry Zurich (Canton of Zurich), the Psychiatric Hospital Malévoz (Monthey, Canton of Valais), and the Hospital of Psychiatry Münsingen (Canton of Bern). The study included all patients with sufficient verbal communication necessary to understand the questionnaire and give informed consent.

A study nurse instructed the patients how to complete the questionnaire. The anonymization of the patient questionnaires took place after entering the data. HCP (mental health nurses, physicians, and psychologists) completed the questionnaire anonymously during working hours. NOK were contacted by mail, or directly if one of their relatives was hospitalized in one of the 3 clinics during the study period. The NOK questionnaire was sent to NOK and additionally asked for age and relationship to the treated relative. Due to the anonymization, a direct connection of NOK questionnaires to patient questionnaires was not possible.

The cantonal Ethics Commission Bern (Ref.-nr. KEK-BE: 2015-00074) reviewed and approved the study. This approval was binding for all survey sites.

### Sample

The study was carried out among patients, NOK, and HCP on psychiatric acute wards of the 3 psychiatric hospitals mentioned above using unselected samples. Overall, data from 1,037 study participants was included. A minority of the participating patients was compulsory admits (20.6%).

Of the NOK, 38.2% were parents of the patients (*N* = 84), 9.5% were children of the patients (*N* = 21), 13.2% were siblings (*N* = 29), 22.3% partners (*N* = 49), 16.8% other related persons (*N* = 37), and 10 were missing this specification. The HCP group consisted of 66.4% nurses (*N* = 243), 25.1% physicians (*N* = 92), and 8.5% psychologists (*N* = 31). There were 6 HCP responses that lacked specific occupational data. For more details, see Table 1.

**Table 1:** Sample.

	***N* (total)**	***N* (Mu)**	***N* (Mo)**	***N* (Zu)**	**Age (ys)**	**SD**	**Female (%)**
Patients	435	97	236	102	40.7	13.3	46.1
HCP	372	146	114	112	37.6	11.7	60.4
NOK	230	99	63	68	49.3	16.1	58.4
Total	1,037	342	413	282	42.0	14.1	53.9

*Mu, Psychiatric Hospital of Muensingen; Mo, Psychiatric Hospital of Monthey; Zu, University Hospital of Psychiatry Zurich; HCP, health care professionals; NOK, next of kin*.

### ACMQ

The Attitudes to Containment Measures Questionnaire (ACMQ) is a self-rating paper-and-pencil questionnaire that has been validated (38) and used in several publications from different countries, and thus from different cultures (24, 25, 28, 30, 39, 48–55). One disadvantage of the ACMQ is that it also collects data on coercive measures that are uncommon or not used at all in Switzerland, such as the net bed.

The 11 main items of the ACMQ have a uniform structure. The specific coercive measure is briefly described and illustrated by a picture, then the participant of the study is asked how acceptable the measure is on a 5-point Likert scale [strongly agree (0) to strongly disagree (5)]. A high value means a high rejection or, respectively, a low acceptance. For each item, the patients were also asked whether they had already experienced this measure. HCP study participants were asked if they had already executed the specific containment measure. In NOK, we inquired as to whether this measure had been administered to their kin. The ACMQ encompasses the following coercive measures: PRN medication, physical restraint, intermittent observation, seclusion, time-out, compulsory intramuscular medication, psychiatric intensive care, mechanical restraint, constant observation, net bed, and open area seclusion.

### Statistical analyses

The statistical analysis was done using SPSS Version 24. Statistical analyses were carried out using standard procedures. Arithmetic means of items were compared using *t*-test for independent samples. The test results were checked for multiple testing by Bonferroni corrections, and the quantification of the differences was determined by effect sizes. In this connection, the pooled standard deviations of the respective group results were taken into account. Due to missing data, there were minor deviations of the number of questionnaires in individual analyses.

Regarding patients, it was distinguished whether they had experienced a coercive measure themselves. Furthermore, the results of compulsory admitted patients were compared to patients treated on a voluntary basis. Whether a patient had never experienced or had experienced at least one of the highly restrictive coercive measures in the past would cause differences in their rating of the measures.

## Results

### Comparing the results of patients, HCP and NOK

The group analysis showed a notable trend. The degree of rejection of all measures was higher among patients than NOK, and higher among NOK than HCP. The general pattern of which coercive measure was rejected the most did not differ between the three groups. All groups rejected the net bed the most, all groups ranked mechanical restraints as the second most unfavorable measure, with seclusion as the third. The biggest difference with respect to the ranking was seen in regards to compulsory intramuscular medication. While it was ranked as the fourth most unfavorable measure by patients and NOK, it was ranked eighth by HCP.

**Figure 1:**
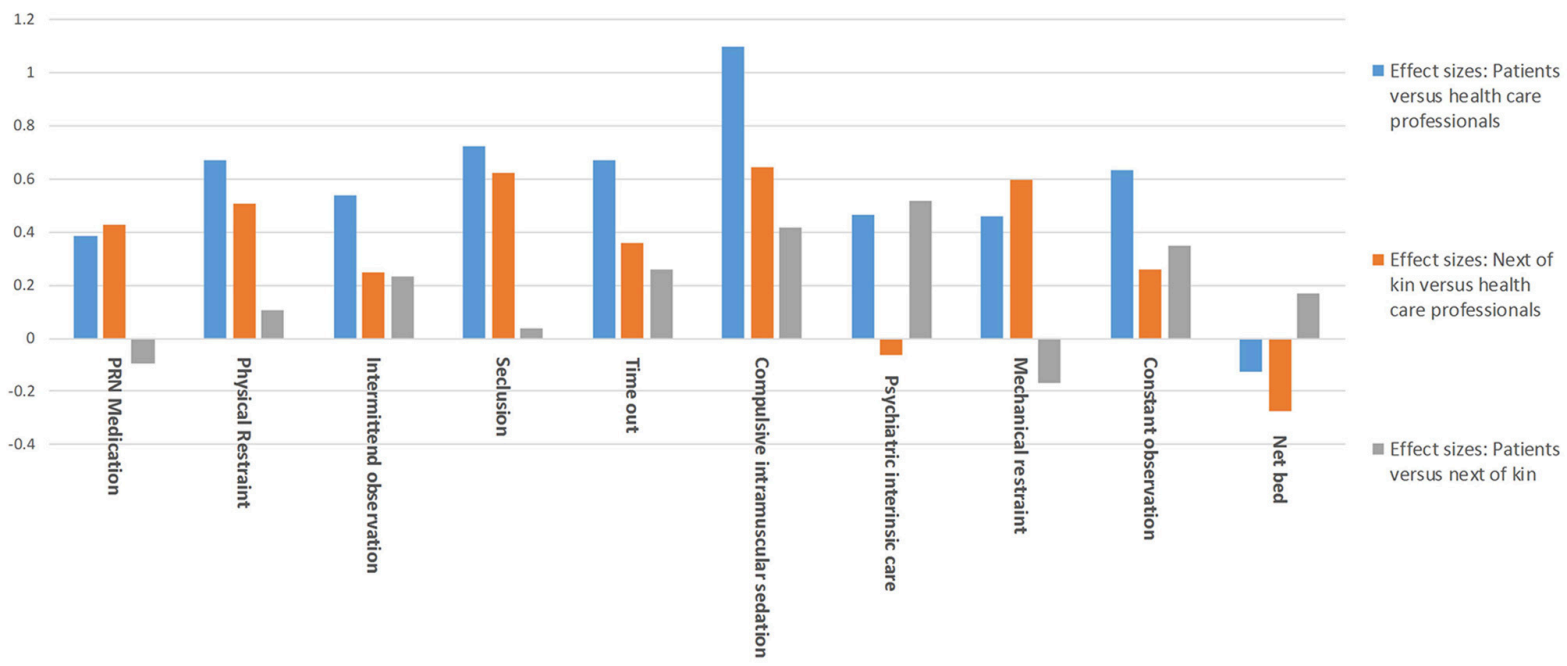
Effect sizes: comparing ratings of patients, health care professionals, and next of kin.

This result was confirmed by analysis of the quantitative differences in the assessments of the individual measures between the groups. Compulsory intramuscular medication produced the largest effect size and thus the largest differences in direct comparison between HCP and patients and between HCP and NOK. The comparison between NOK and HCP also shows that seclusion and mechanical restraint are rated differently. Acceptance of treatment on a locked acute ward is the only measure that shows no significant difference between HCP and NOK. The differences between NOK and patients are small on average: A medium effect size was found only for treatment on an acute closed ward, as NOK rated this measure as significantly more acceptable (see Table 2; Figure 1).

**Table 2:** Attitudes toward containment measures: mean ratings of patients, health care professionals, and next of kin.

	**Patient (*****N*** = **435)**		**Health care professionals (*****N*** = **372)**		**Next of kin (*****N*** = **230)**		**Patients vs. health care professionals**		**Patients vs. next of kin**		**Health care professionals vs. next of kin**	
	**Mean**	**SD**	**Mean**	**SD**	**Mean**	**SD**	***t***	***p*-corr**	***t***	***p*-corr**	***t***	***p*-corr**
Pro re nata medication	1.81	0.99	1.30	0.52	1.91	0.89	9.31	< 0.001	−1.32	n.s.	−9.46	< 0.001
Physical restraint	2.74	1.27	2.12	0.90	2.65	1.03	8.05	< 0.001	1.02	n.s.	−6.35	< 0.001
Intermittent observation	2.04	0.99	1.36	0.60	1.76	0.65	11.78	< 0.001	4.33	< 0.001	−7.63	< 0.001
Seclusion	3.08	1.31	2.49	1.16	3.05	1.09	6.74	< 0.001	0.35	n.s.	−5.83	< 0.001
Time-out	2.36	1.14	1.66	0.77	2.10	0.84	10.37	< 0.001	3.33	0.011	−6.59	< 0.001
Compulsive intramuscular sedation	3.07	1.34	2.03	0.76	2.72	1.08	13.70	< 0.001	3.55	0.005	−8.52	< 0.001
Psychiatric interinsic care	2.69	1.21	2.29	1.14	2.24	1.10	4.75	< 0.001	4.82	< 0.001	0.57	n.s.
Mechanical restraint	3.51	1.27	3.13	1.18	3.65	1.14	4.34	< 0.001	−1.42	n.s.	−5.26	< 0.001
Constant observation	2.40	1.08	1.72	0.80	2.04	0.85	10.17	< 0.001	4.70	< 0.001	−4.60	< 0.001
Net bed	4.10	1.11	4.22	0.94	3.94	1.00	−1.71	n.s.	1.87	n.s.	3.51	0.006
Open area seclusion	2.54	1.10	2.47	1.12	2.21	0.85	0.87	n.s.	4.28	< 0.001	3.23	0.016
Mean (all items)	2.76	0.75	2.26	0.48	2.57	0.57	11.51	< 0.001	3.66	0.003	−7.00	< 0.001

*n.s., not significant; p-corr, p-values are Bonferroni corrected*.

### Subanalyses of the patient sample

Patients admitted on a compulsory basis tended to rate coercive measures as less acceptable than voluntarily hospitalized patients. The strongest effect sizes were found for physical restraint and compulsory intramuscular medication (see Table 3).

**Table 3:** Comparing ACMQ ratings of voluntary vs. compulsorily admitted patients.

	**Voluntary admitted (*****N*** = **352, 81.1%)**		**Complusorily admitted (*****N*** = **82, 18.9%)**		**Statistics**			
	**Mean**	**SD**	**Mean**	**SD**	***t***	***p***	***p*-corr**	**ES**
Pro re nata medication	1.74	0.94	2.14	1.12	−2.97	0.004	0.044	0.39
Physical restraint	2.61	1.21	3.32	1.38	−4.28	< 0.001	< 0.001	0.55
Intermittent observation	1.99	0.95	2.25	1.15	−1.89	n.s.	n.s.	0.25
Seclusion	3.07	1.31	3.15	1.33	−0.50	n.s.	n.s.	0.06
Time-out	2.31	1.11	2.59	1.24	−1.97	0.049	n.s.	0.23
Compulsive intramuscular sedation	2.95	1.31	3.57	1.35	−3.77	< 0.001	0.002	0.46
Psychiatric interinsic care	2.67	1.18	2.78	1.29	−0.70	n.s.	n.s.	0.08
Mechanical restraint	3.43	1.27	3.86	1.20	−2.79	0.006	n.s.	0.35
Constant observation	2.34	1.02	2.68	1.26	−2.23	0.028	n.s.	0.29
Net bed	4.04	1.14	4.34	0.98	−2.20	0.028	n.s.	0.28
Open area seclusion	2.45	1.03	2.90	1.27	−2.99	0.003	0.042	0.39
Mean (all items)	2.69	0.73	3.06	0.78	−4.08	< 0.001	0.001	0.49

*ACMQ, Attitudes to Containment Measures Questionnaire; n.s., not significant; p-corr, p-values are Bonferroni corrected*.

Patients who experienced at least one strongly restricting coercive measure (physical restraint, seclusion, compulsory intramuscular medication or mechanical restraint; *N* = 38, 34.7%) rated the coercive measures as less acceptable compared to patients who had not experienced coercion (*t* = 3.15, *p* = 0.002). The effect size (ES) of this difference was 0.33. Significantly higher rejections were found for PRN medication (*t* = 2.29, *p* = 0.023, ES 0.26.), physical restraint (*t* = 3.14, *p* = 0.002, ES 0.32), compulsory intramuscular medication (*t* = 2.89, *p* = 0.004, E. 0.31), mechanical restraint (*t* = 2.10, *p* = 0.037, ES 0.22) and the network bed (*t* = 2.36, *p* = 0.019, ES = 0.25). After the Bonferroni correction, only physical restraint and coercive medication were statistically significant. If this analysis is limited in line with Dack et al. (2) to patients who had experienced a compulsory intramuscular medication, a virtually identical result is obtained (mean value of all measures *t* = 2.98, *p* = 0.003, ES = 0.35).

## Discussion

According to our hypothesis, patients and NOK consistently rejected all coercive measures more strongly than HCP. The latter presumably consider the potential benefits of these measures more often and feel responsible for preventing harm to other patients and themselves. The low values for HCP may also be seen as a justification for their own behavior.

When viewing the ranking of the ratings over the absolute assessment values of the measures, all three study groups show an identical ranking order for the three items with the highest rating. In line with several publications (29, 30, 54), Swiss patients, NOK, and HCP most clearly rejected the net bed. This measure is not applied in Switzerland and is likely, as in Finland, perceived as “inhumane and cruel” (28). Mechanical restraints and seclusion were rejected second- and third-most by all groups. In contrast to the net bed, these measures are widely used in German-speaking countries. Patients preferred pro re nata medication, physical restraint, psychiatric intensive care and constant observation to compulsive intramuscular injection. In some situations, intramuscular medication may be difficult to avoid. However, in respect to the results of our study, clinicians should evaluate whether less aversive measures, such as pro re nata medication, psychiatric intensive care and constant observation can be used in its place. In other cases, injection might be prevented by steps, such as changing the culture or atmosphere of the ward. Notably, compulsive intramuscular injection was preferred by the patients to mechanical restraint. These two measures are often combined in clinical practice. Clinicians should in these cases also evaluate whether mechanical restraint can be at least avoided by applying compulsory injection only.

The largest rating differences between HCP and patients surround compulsory intramuscular injection of medication. With an effect size >1, this difference may be related to the conviction of HCP that intramuscular injection of medication is therapeutically necessary (5). The focus of HCP on applying treatment interventions rather than mere security measures explains these beliefs. Still, there is a risk that HCP use these measures with a relatively low threshold and thus insufficiently consider their negative effects, namely the deterioration of the therapeutic relationship due to disapproval of coercion by patients (37). Additionally, the therapeutic effect of a compulsory medication could not be verified by evidence (26), and that medication is frequently used for temporary control of behavior (3).

In accordance with Dack et al. (38), we observed that patients who were medicated against their will had more negative attitudes toward all coercive measures. The differing ratings of the patients, as well as the ratings of all measures taken together, show that such an act has high costs. Therefore, HCP must avoid this intervention whenever possible.

More than a third of all coercive measures are triggered by a HCP-patient interaction (47). Consequently, the use of compulsory medication is preventable in advance. Solutions may include greater sensitivity to the use of informal coercion, which may represent a precursor of coercive measures and lead to disruptions in the therapeutic relationship (56). Reducing the consequences of coercive measures could include debriefing of events, which in Switzerland is considered standard (40) (SAMW guideline). Clinicians should actively seek patient perspectives on compulsory medication retroactively to minimize secondary negative impact. The reduction of perceived coercion should always be an objective while administering coercive measures to reduce negative effects within the therapeutic relationship. Clinicians can achieve this through transparent communication, choices concerning coercive measures, sound justification for these measures, and respect for the patients' perspectives (31, 57).

Patients who were admitted compulsorily were more likely to show a negative attitude toward coercive measures, which concurs with numerous publications (5, 12). The main reason for these negative attitudes may be the acute illness of compulsory patients at the beginning of their treatment (19). Unfortunately, our results could not differentiate whether patients had a negative attitude before beginning their treatment, if a negative stance to psychiatric treatment causes their negative attitude, or if they acquired their attitude after experiencing involuntary admission.

In general, NOK reject coercive measures significantly less strongly than do patients. This result is consistent with Ranieri et al. (41). This likely stems from NOK approval toward coercive treatment conducted for the wellbeing of their relatives. They often find themselves caught in an ambivalent position, as they simultaneously want to avoid patient suffering from restricted autonomy and freedom of movement. In absolute terms, however, NOK displayed ratings much closer to those of patients. It is noteworthy that the treatment on the locked acute ward is the only item that did not differ between NOK and HCP while also showing a significant difference between NOK and patients. Taken together, although in favor of treatment on the acute ward, NOK are critical of the concrete measures. In other words, they agree that their relatives must be treated, but not on the methods of treatment. Considering the essential role of NOK in patient care, especially of seriously ill psychiatric patients, it is vital to actively include NOK after administering coercive measures (58).

In summary, we can conclude that our study has several limitations and strengths. One such shortcoming is the relatively low participation rates of NOK and patients. In particular, patients treated with coercive measures often refused participation in the study. Further, data collection was done after stabilization; however, it did occur during treatment on acute wards. It is possible that residual symptoms and the patient's own experiences influenced the results. The main strength of our study is, according to our literature review, that it is the first to examine ratings of containment measures by NOK, HCP, and patients comparatively. In addition, the large sample allows for high statistical power and the detection of medium effects.

## Author contributions

PM: translation, editing, and writing; SB: research, data collection, and writing; TR and MJ: research design, data analysis, and writing; GK and PP: data collection, writing, and analysis; EB and FH: data collection and analysis.

### Conflict of interest statement

The authors declare that the research was conducted in the absence of any commercial or financial relationships that could be construed as a potential conflict of interest.
